# Neuroleptic Malignant Syndrome on Chronic Trifluoperazine Therapy: A Diagnostic Dilemma

**DOI:** 10.1002/ccr3.71631

**Published:** 2025-12-05

**Authors:** Sagun Baral, Sushmita Bhattarai, Khem Chandra Joshi

**Affiliations:** ^1^ Department of Internal Medicine Rapti Academy of Health Sciences Dang Nepal

**Keywords:** antipsychotics, creatine kinase, encephalopathy, neuroleptic malignant syndrome, trifluoperazine

## Abstract

Neuroleptic malignant syndrome (NMS) is a rare, life‐threatening reaction to antipsychotic medication. This report describes the case of a 62‐year‐old male with chronic trifluoperazine use who presented with fever, rigidity, and altered mental status. Although sepsis or meningoencephalitis was initially suspected, an elevated creatine kinase and fulfillment of Levenson's criteria confirmed the diagnosis of NMS. The patient's condition improved following supportive care, administration of bromocriptine, and lorazepam. This case highlights the diagnostic challenges of NMS in patients on a stable antipsychotic regimen and underscores the importance of early recognition, particularly after excluding infectious etiologies.

## Key Clinical Message

Neuroleptic malignant syndrome should be considered in the differential diagnosis for febrile, encephalopathic patients receiving antipsychotics, even on a stable dosage. The presence of cytopenias may initially suggest an infectious etiology. Early recognition and multidisciplinary management are critical for improving patient outcomes.

## 1 Introduction

Neuroleptic malignant syndrome (NMS), first described by Delay et al., is a neurological emergency representing a severe adverse reaction to neuroleptic medications [1]. While its incidence is low at approximately 0.2% among neuroleptic users, a high index of suspicion is required due to its potentially fatal course, with reported mortality rates ranging from 3.3% to 27.7% [1]. The syndrome is classically characterized by severe muscular rigidity and hyperthermia, accompanied by autonomic dysfunction (profuse sweating, labile heart rate, and blood pressure), altered mental status, and laboratory abnormalities such as leukocytosis and elevated creatine phosphokinase (CPK) levels [2]. Established risk factors include the use of high‐potency first‐generation antipsychotics, dehydration, exhaustion, malnutrition, parenteral administration, rapid dose escalation, and a prior history of NMS [3]. Although most cases develop within the first four weeks of initiating antipsychotics—two‐thirds within the first week—NMS can also manifest in patients on long‐term, stable‐dose treatment [2]. This report presents the case of a 62‐year‐old male with a long‐standing psychiatric illness, maintained on a stable regimen including trifluoperazine, who presented with fever, generalized rigidity, and altered mental status. The initial clinical picture was confounded by fever, thrombocytopenia, and leukopenia, suggesting an infectious etiology such as meningoencephalitis or sepsis. This case underscores that NMS can occur in patients on chronic, stable‐dose antipsychotic therapy and highlights the critical need for early recognition and multidisciplinary collaboration, particularly when initial presentations mimic infection.

## 2 Case History/Examination

A 62‐year‐old male with a chronic psychiatric illness, who was under regular outpatient psychiatric care, was brought to the emergency department with a two‐day history of high‐grade fever and a one‐day history of poor oral intake and altered mental status. His precise psychiatric diagnosis was unavailable due to a lack of accessible records. He had been on a stable psychotropic regimen for several months, which included trifluoperazine (5 mg), trihexyphenidyl (2 mg), chlordiazepoxide (10 mg), amitriptyline (25 mg), donepezil (5 mg), memantine (10 mg), divalproex sodium (500 mg), and fluvoxamine (50 mg).

On examination, the patient was febrile (temperature: 103°F), hypotensive (blood pressure: 90/60 mmHg), and tachycardic (pulse: 121 bpm). His respiratory rate was 18 breaths/min with an oxygen saturation of 95% on room air. Neurological assessment revealed a Glasgow Coma Scale (GCS) score of 10 (E4V1M5). Pupils were equal and reactive to light. He demonstrated generalized rigidity in all four limbs with increased muscle tone and bilateral flexor plantar responses. The presence of neck stiffness raised initial suspicion of meningoencephalitis.

## 3 Differential Diagnosis, Investigations, and Treatment

Empirical antibiotics (ceftriaxone and doxycycline) were initiated. A lumbar puncture was performed, and cerebrospinal fluid (CSF) analysis was within normal limits (Table 1). CSF cultures, Gram stain, and PCR studies for bacterial and viral pathogens were negative. Dengue IgM and NS1 antigen, as well as scrub typhus IgM, were negative. Blood and urine cultures were sterile. CRP and procalcitonin were within normal ranges.

**TABLE 1:** Cerebrospinal fluid (CSF) analysis on Day 0.

Parameter	Result	Reference range
Total cell count (/mm^3^)	< 3	0–5
Protein (mg/dL)	43.1	15–45
Glucose (mg/dL)	74	40–80
Adenosine deaminase (U/L)	1.3	< 9
Culture and Gram stain	Sterile	—

Initial laboratory investigations revealed normocytic normochromic anemia (Hb: 12.1 g/dL), leukopenia (TLC: 3740/μL), and severe thrombocytopenia (platelet count: 42,000/μL). Liver function tests showed elevated transaminases (AST 173 U/L, ALT 74 U/L), with a total bilirubin of 0.84 mg/dL and direct bilirubin of 0.35 mg/dL. Renal parameters were within normal limits. Arterial blood gas analysis showed metabolic alkalosis (pH: 7.465, bicarbonate: 31.4 mmol/L, lactate: 0.6 mmol/L). Non‐contrast CT of the head revealed cerebral atrophy, with moderately dilated sylvian fissures and ventricles, but no acute pathology.

Despite empiric antibiotics and supportive care, the patient's clinical condition deteriorated over the subsequent 48 h, with worsening rigidity, persistent fever, and a further decline in mental status. In the absence of a confirmed infectious source, NMS was considered. The diagnosis was supported by the fulfillment of Levenson's criteria, including hyperthermia, severe rigidity, altered mental status, autonomic dysfunction (labile blood pressure and tachycardia), and elevated creatine kinase (CK) level [4].

Serum CK was significantly elevated at 1268 U/L on Day 2, with concurrent LDH of 713 U/L. The patient was transferred to the intensive care unit (ICU), where he received aggressive supportive therapy, including intravenous fluids, active external cooling, and close monitoring of renal and cardiovascular functions. Bromocriptine 2.5 mg three times daily and lorazepam 2 mg twice daily were initiated and titrated based on clinical response.

CK decreased to 445 U/L on Day 3, with LDH remaining at 700 U/L. Hematological parameters improved progressively with supportive care, with platelet count rising to 52,000/μL on Day 2 and 118,000/μL on Day 4.

Differential diagnoses were considered and systematically ruled out. Serotonin syndrome was excluded due to the absence of clonus, hyperreflexia, mydriasis, or recent serotonergic drug initiation. The Hunter criteria were not met [5]. Catatonia was considered less likely in the absence of waxy flexibility, echolalia, or mutism before symptom onset, and there was no prior documentation of catatonic episodes. The patient had no history of recent antipsychotic dose escalation or parenteral neuroleptic administration, supporting an atypical presentation.

## 4 Outcome and Follow‐Up

The diagnosis of NMS was made based on clinical evolution, diagnostic criteria, and marked improvement with targeted treatment. By Day 7, rigidity subsided, fever defervesced, and GCS improved to E4V3M6. CK levels normalized, and there were no signs of renal or hepatic decompensation. The patient was gradually weaned off bromocriptine and transferred to the psychiatry unit for further evaluation and long‐term medication review.

This case illustrates an atypical NMS presentation in a patient on a chronic, stable dose of trifluoperazine, without recent medication changes or other evident precipitating factors. The diagnostic process was complicated by leukopenia and thrombocytopenia, which mimicked sepsis and contributed to a delay in diagnosis. This report, therefore, highlights the importance of maintaining a high index of suspicion for NMS, even in patients with nonclassical presentations or complex polypharmacy.

## 5 Discussion

NMS is a rare but life‐threatening complication of antipsychotic therapy that requires prompt diagnosis and management. While classically associated with the initiation or dose escalation of high‐potency first‐generation antipsychotics, this case illustrates that NMS can also develop in patients on long‐term, stable‐dose regimens.

Our patient developed NMS while on a stable dose of trifluoperazine, a deviation from the classic presentation, where up to two‐thirds of cases occur within the first week of initiating neuroleptics, and most within four weeks [6]. However, case reports and retrospective reviews have documented NMS in the context of chronic antipsychotic use, likely triggered by physiological stressors or concurrent medical illness [2, 7]. In our case, a preceding systemic illness or metabolic disturbance may have lowered the threshold for dopamine blockade‐induced neurotoxicity. The extensive polypharmacy in this patient (Table 2) further complicated the diagnostic picture.

**TABLE 2:** Psychotropic medications and potential contributions to NMS.

Medication	Class/mechanism	Potential contribution to NMS
Trifluoperazine	Typical antipsychotic, potent D2 blockade	Central dopamine receptor blockade → core trigger of NMS
Trihexyphenidyl (THP)	Anticholinergic	Anticholinergic load may mask early symptoms, increasing vulnerability
Amitriptyline	Tricyclic antidepressant (serotonergic + anticholinergic)	Anticholinergic and serotonergic effects may potentiate risk
Fluvoxamine	SSRI (serotonergic)	Serotonergic activity—considered in differential diagnosis with serotonin syndrome
Chlordiazepoxide	Benzodiazepine (GABA agonist)	Sedative; not causative, but may blunt rigidity/anxiety
Divalproex sodium	Mood stabilizer, GABAergic	Not directly implicated; polypharmacy increases complexity
Donepezil	Acetylcholinesterase inhibitor	Cholinergic effects; no direct link but contributes to polypharmacy
Memantine	NMDA receptor antagonist	No direct link, but may interact with CNS processes

The clinical picture was further complicated by overlapping features with sepsis and central nervous system (CNS) infections, such as fever, altered mental status, leukopenia, thrombocytopenia, and neck stiffness. This led to an initial diagnostic focus on infectious etiologies, delaying consideration of NMS. However, the lack of an identifiable pathogen, normal CSF analysis, and failure to respond to broad‐spectrum antibiotics prompted reconsideration of the diagnosis. Elevated CK, persistent rigidity, autonomic instability, and altered sensorium—hallmarks of NMS—fulfilled the Levenson diagnostic criteria [4]. The timeline of events is depicted in Table 3.

**TABLE 3:** Clinical timeline of events.

Day	Clinical events and key findings	Interventions
Day 0	Fever (103°F), altered sensorium, rigidity, neck stiffness. Labs: leukopenia, thrombocytopenia (42,000/μL). CSF normal	Empiric antibiotics (ceftriaxone, doxycycline), supportive care
Day 1	Persistent fever, worsening rigidity	Continued antibiotics, monitoring
Day 2	Further decline in mental status, CK elevated (1268 U/L). Levenson's criteria fulfilled	Diagnosis of NMS made. Transferred to ICU. Started bromocriptine and lorazepam
Day 3	CK decreased to 445 U/L, and platelet count improved	Supportive care continued
Day 4	Platelet count 118,000/μL, clinical improvement	Continued therapy
Day 7	Rigidity resolved, fever subsided, GCS improved to E4V3M6	Gradual tapering of bromocriptine, transfer to the psychiatry unit

This case underscores the diagnostic complexity of NMS in polypharmacy settings. The patient was on several centrally acting agents, including anticholinergics, antidepressants, benzodiazepines, mood stabilizers, and cognitive enhancers. Although serotonin syndrome was considered due to the use of fluvoxamine and amitriptyline, the clinical features did not meet the Hunter criteria, particularly in the absence of hyperreflexia, clonus, or mydriasis [5]. Catatonia was also considered but ruled out based on the absence of classic signs such as waxy flexibility, echolalia, and negativism.

The patient's management aligned with established NMS treatment protocols [8]. The discontinuation of trifluoperazine, combined with aggressive supportive care, a dopamine agonist (bromocriptine), and a benzodiazepine (lorazepam), resulted in a rapid decline in CK levels and steady clinical improvement. He was not rechallenged with antipsychotics during this admission. At discharge, he remained under psychiatric follow‐up for careful review of his psychotropic regimen and long‐term management, with plans to avoid high‐potency neuroleptics and to monitor closely for early warning signs of recurrence.

The pathophysiology of NMS is primarily attributed to central dopamine receptor blockade in the hypothalamus and nigrostriatal pathways, leading to dysregulated thermoregulation and severe muscle rigidity [8]. However, individual susceptibility appears to vary and may be modulated by genetic, metabolic, and pharmacologic factors, especially in elderly patients or those with underlying neurological vulnerabilities [9].

This case demonstrates that NMS can develop even in patients on long‐term, stable‐dose antipsychotic therapy. The clinical presentation, which may include systemic features like fever, cytopenias, and altered mental status, may mimic infectious or other neurological emergencies, contributing to diagnostic delays. Therefore, clinicians should maintain a high index of suspicion for NMS in any febrile patient on neuroleptics who presents with rigidity and encephalopathy, irrespective of treatment duration. Prompt discontinuation of the offending agent, alongside the initiation of supportive and targeted pharmacologic therapies, is essential for improving outcomes. Effective management of this life‐threatening condition often necessitates a multidisciplinary approach.

## Author Contributions

**Sagun Baral:** conceptualization, data curation, formal analysis, methodology, supervision, validation, writing – original draft, writing – review and editing. **Sushmita Bhattarai:** conceptualization, formal analysis, investigation, supervision, writing – review and editing. **Khem Chandra Joshi:** methodology, supervision, visualization, writing – review and editing.

## Funding

The authors have nothing to report.

## Ethics Statement

Ethical approval was not required for this single‐patient case report in accordance with institutional guidelines.

## Consent

Written informed consent was obtained from the patient for publication of this case report and accompanying data.

## Conflicts of Interest

The authors declare no conflicts of interest.

## Data Availability

The data that support the findings of this study are available from the corresponding author upon reasonable request.

## References

[1] S. Modi , D. Dharaiya , L. Schultz , and P. Varelas , “Neuroleptic Malignant Syndrome: Complications, Outcomes, and Mortality,” Neurocritical Care 24, no. 1 (2016): 97–103, 10.1007/s12028-015-0162-5. 26223336

[2] R. Velamoor , “Neuroleptic Malignant Syndrome: A Neuro‐Psychiatric Emergency—Recognition, Prevention, and Management,” Asian Journal of Psychiatry 29 (2017): 106–109, 10.1016/j.ajp.2017.05.004. 29061403

[3] M. R. Ware , D. B. Feller , and K. L. Hall , “Neuroleptic Malignant Syndrome,” Primary Care Companion CNS Disorders 20, no. 1 (2018): 17r02185, 10.4088/PCC.17r02185. 29325237

[4] J. L. Levenson , “Neuroleptic Malignant Syndrome,” American Journal of Psychiatry 142, no. 10 (1985): 1137–1145, 10.1176/ajp.142.10.1137. 2863986

[5] E. J. C. Dunkley , G. K. Isbister , D. Sibbritt , A. H. Dawson , and I. M. Whyte , “The Hunter Serotonin Toxicity Criteria: Simple and Accurate Diagnostic Decision Rules for Serotonin Toxicity,” QJM 96, no. 9 (2003): 635–642, 10.1093/qjmed/hcg109. 12925718

[6] J. R. Strawn , P. E. Keck, Jr. , and S. N. Caroff , “Neuroleptic Malignant Syndrome,” American Journal of Psychiatry 164, no. 6 (2007): 870–876, 10.1176/ajp.2007.164.6.870. 17541044

[7] L. Tse , A. M. Barr , V. Scarapicchia , and F. Vila‐Rodriguez , “Neuroleptic Malignant Syndrome: A Review From a Clinically Oriented Perspective,” Current Neuropharmacology 13, no. 3 (2015): 395–406, 10.2174/1570159X13999150424113345. 26411967 PMC4812801

[8] A. L. Pelonero , J. L. Levenson , and A. K. Pandurangi , “Neuroleptic Malignant Syndrome: A Review,” Psychiatric Services 49, no. 9 (1998): 1163–1172, 10.1176/ps.49.9.1163. 9735957

[9] D. Berardi , M. Amore , P. E. Keck, Jr. , M. Troia , and M. Dell'Atti , “Clinical and Pharmacologic Risk Factors for Neuroleptic Malignant Syndrome: A Case‐Control Study,” Biological Psychiatry 44, no. 8 (1998): 748–754, 10.1016/S0006-3223(97)00530-1. 9798079

